# THE COMBINED EFFECTS OF SOCIAL ISOLATION AND LONELINESS ON PSYCHOLOGICAL WELL-BEING DURING THE COVID-19 PANDEMIC

**DOI:** 10.1093/geroni/igac059.1261

**Published:** 2022-12-20

**Authors:** Ke Li, Fengyan Tang

**Affiliations:** University of Pittsburgh, Pittsburgh, Pennsylvania, United States; University of Pittsburgh, Pittsburgh, Pennsylvania, United States

## Abstract

Social isolation and loneliness are related but distinct constructs. A number of studies have examined these two constructs separately; however, the combined and interactive effects of social isolation and loneliness on health outcomes have rarely been studied. Using the most recent data of the Health and Retirement Study (HRS 2020) collected during the pandemic, this study aimed to explore the latent classes of social isolation and loneliness among adults aged 60 and older (N=3,486) and to examine the associated psychological well-being. Social isolation was measured by five indicators, including living alone, no membership in any organizations, and less than once a month contact with children, relatives, and friends. Loneliness was measured by the 3-item UCLA scale. Four classes were identified by the Latent Class Analysis (LCA): neither isolated nor lonely (class 1, 35%), living alone and lonely (class 2, 25%), no social participation and lonely (class 3, 18%), and highly isolated and lonely (class 4, 23%). The results of multivariate regression indicated that compared to respondents who were neither isolated nor lonely, those who were in the class of living alone and lonely and the class of highly isolated and lonely had more depressive symptoms, stress, anxiety, worry, loneliness during the pandemic. The latent class of no social participation and lonely was associated with more depressive symptoms and covid-related stress. This study emphasizes the importance of specialized intervention strategies targeting the unique needs of older adults with different experiences of social isolation and loneliness.

